# Supplementary material for: Adhesion to nanofibers drives cell membrane remodeling through one-dimensional wetting
Source: Nat Commun. 2018 Oct 25;9:4450. doi: 10.1038/s41467-018-06948-x (PMC6202395; doi:10.1038/s41467-018-06948-x)
Supplement: Supplementary file 1 — Supplementary Information [file 41467_2018_6948_MOESM1_ESM.pdf]

Supplementary Information

Adhesion to nanofibers drives cell membrane remodeling through one-dimensional wetting

Charles-Orszag *et al*.

Supplementary Figure 1

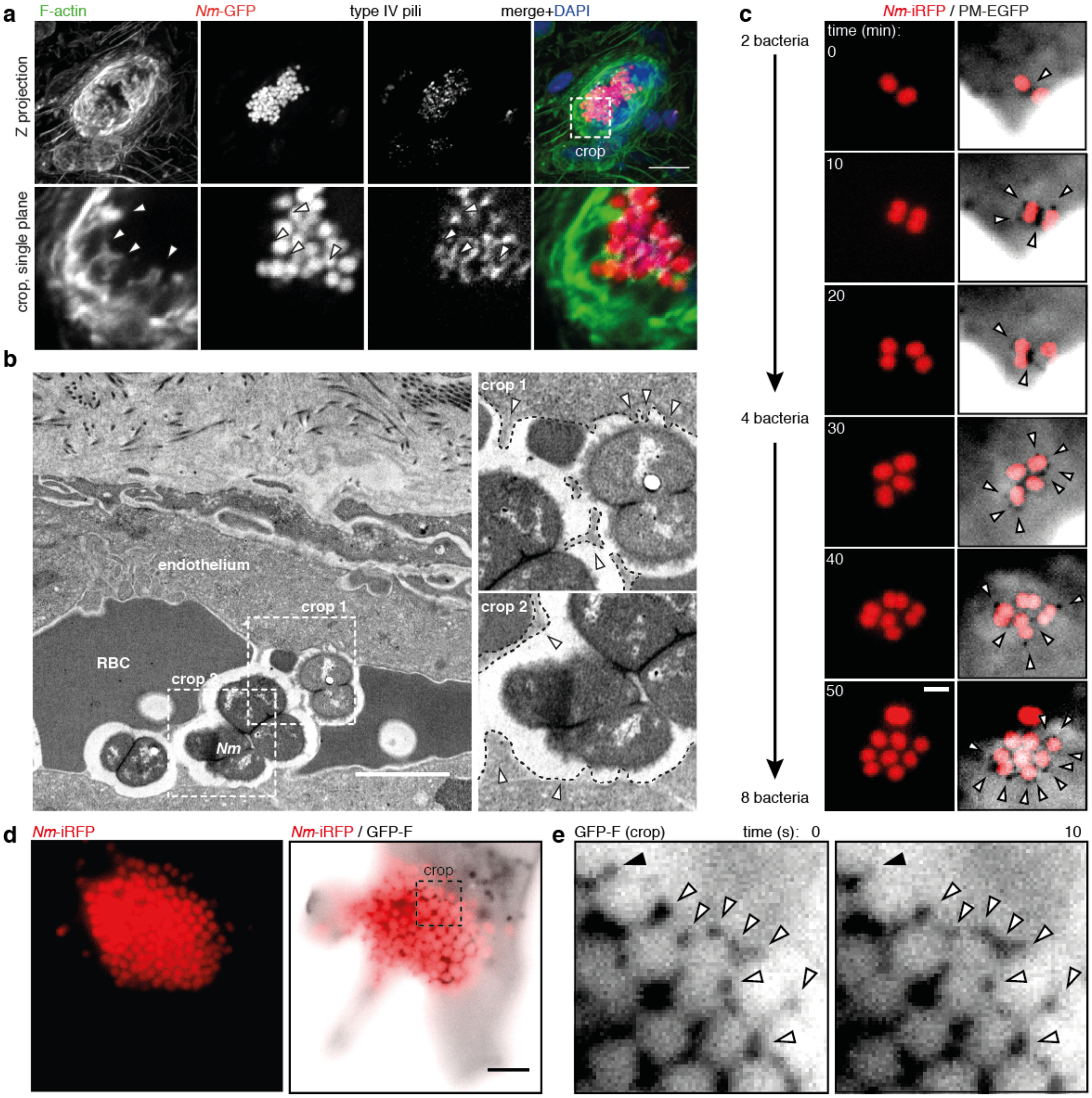

Supplementary Figure 1. Further characterization of plasma membrane protrusions induced by *Nm in vivo* and by individual bacteria *in vitro*. (a) Histoimmunolabeling of human blood vessels in a mouse after 3h of infection with *N. meningitidis*(*Nm*)-GFP showing plasma membrane protrusions containing F-actin (arrowheads). Scale bar, 10 µm. Representative of n=2 mice. (**b**) Transmission electron micrograph showing the plasma membrane of endothelial cells (dashed lines in the crops) remodeled beneath and between aggregated bacteria in an infected human vessel (arrowheads). Scale bar, 2 µm. n=1. (**c**) Oblique illumination live imaging of a micropatterned endothelial cell expressing the plasma membrane marker PalmitoylMyristoyl-EGFP (PM-EGFP, inverted contrast) infected by individual *Nm*-iRFP. Plasma membrane protrusions initiated at the level of two bacteria are accumulated as bacteria divide on the host cell surface and remain within the nascent microcolony (arrowheads). Scale bar, 2 µm. Representative of n=3 experiments. (**d**-**e**) Oblique illumination live imaging of an endothelial cell expressing the membrane marker GFP-F infected by an aggregate of *Nm*-iRFP shows that plasma membrane protrusions in this case are no longer dynamic (white arrowheads) with rare events of disappearing protrusions occurring at the edge of the bacterial aggregate (black arrowhead). Representative of n=3 experiments. Scale bar, 5 µm.

Supplementary Figure 2

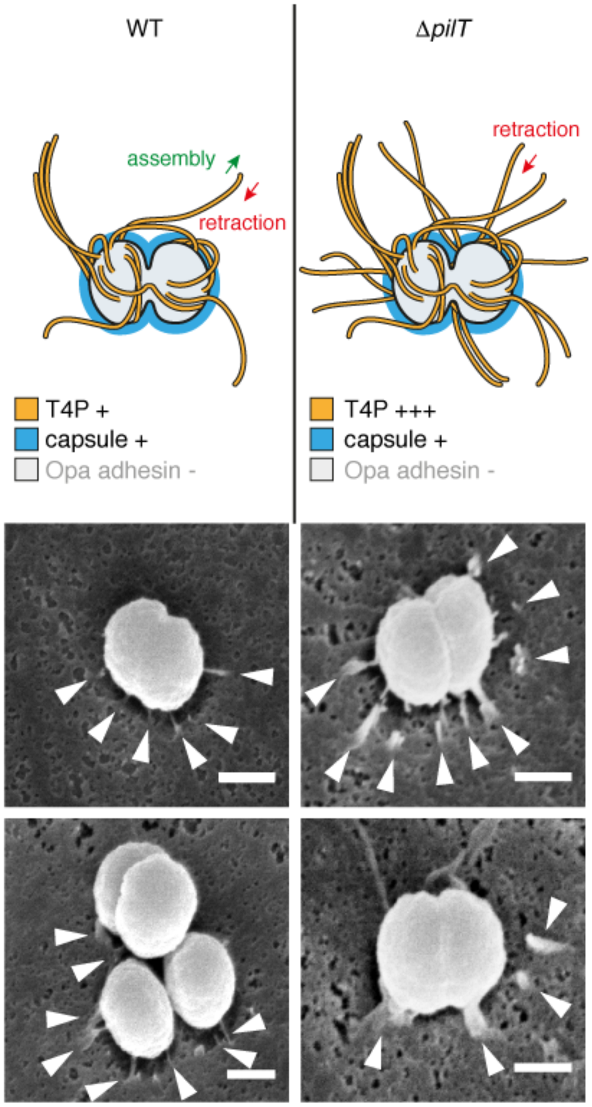

Supplementary Figure 2. T4P retraction-deficient individual bacteria trigger plasma membrane protrusions. Scanning electron microscopy showing that the *pilT* mutant, which cannot retract T4P, still induces plasma membrane remodeling in the form of discrete protrusions as in the wild-type strain (WT). However, either more protrusions or protrusion slightly larger are observed, likely due to the higher amount of T4P produced by the *pilT* strain. Note that T4P are not visible in this preparation. Scale bars, 500 nm. n=1 experiment.

Supplementary Figure 3

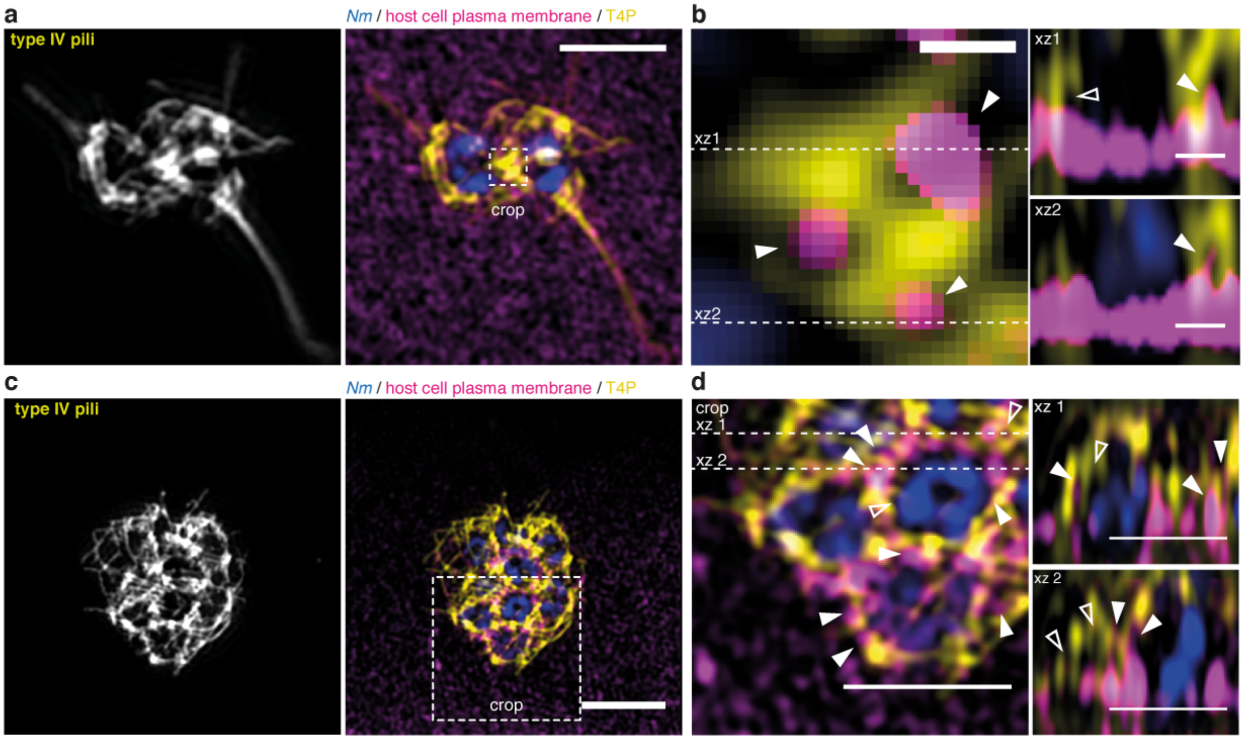

Supplementary Figure 3. T4P-plasma membrane interface visualized by 3D SIM. (**a**) 3D Structure Illumination Microscopy (SIM) micrographs of a pair (**a**, **b**) and a small aggregate of 8 (**c**, **d**) meningococci (DAPI, blue) after 30 min infection of endothelial cells expressing the membrane marker PM-EGFP and immunostained for T4P and GFP. (**a**) and (**c**) show T4P detection and merged images in Z projections. Scale bars, 2 µm and 3 µm. (**b**) and (**d**) are cropped merged images and Z-sections showing details of T4P organization with empty spaces between fibers (empty arrowheads) and spaces occupied by plasma membrane protrusions (filled arrowheads). Scale bars in (**b**), 200 nm for the first inset then 500 nm. Scale bars in (**d**), 2 µm. n=3 experiments.

Supplementary Figure 4

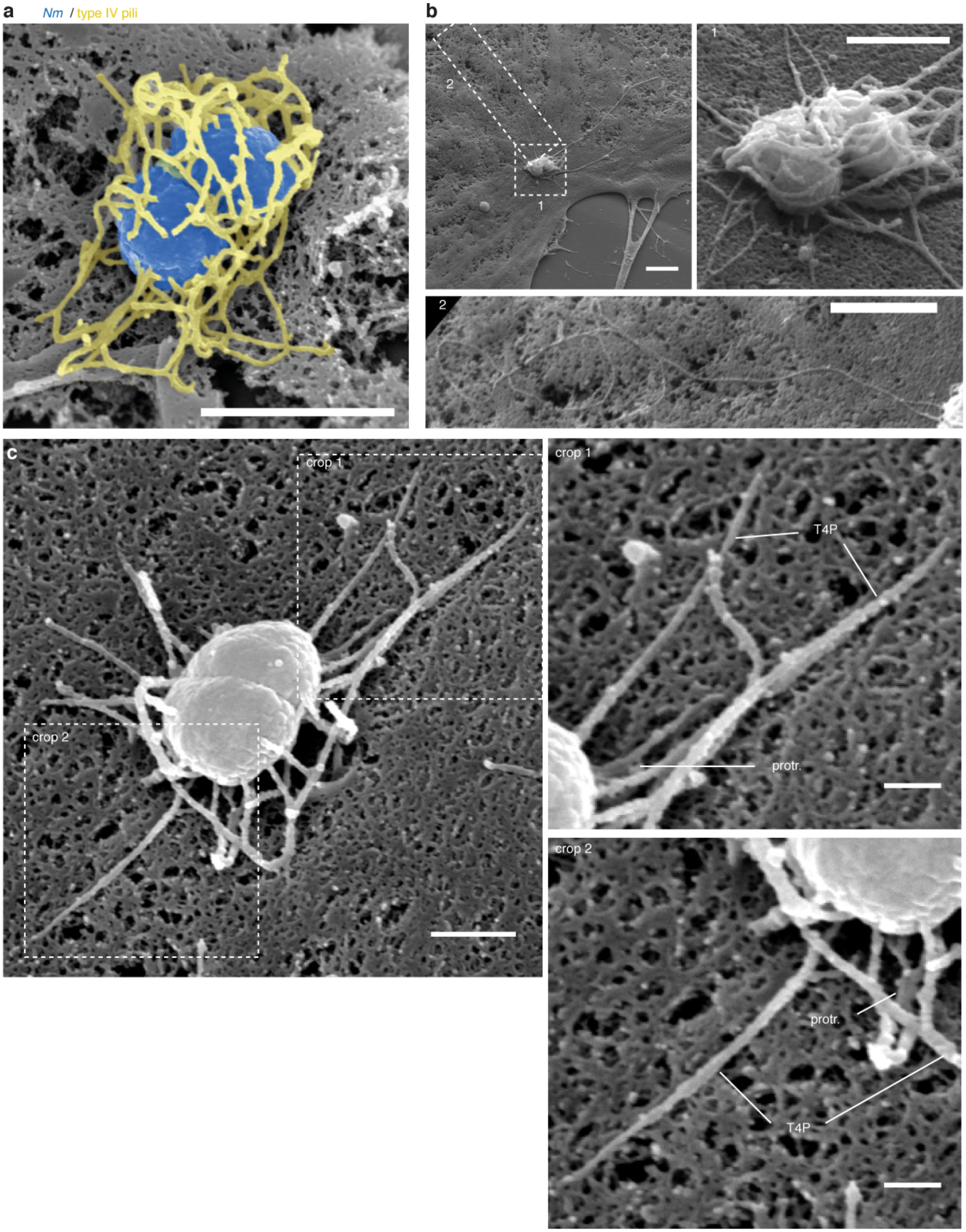

Supplementary Figure 4. Additional examples of *Nm* T4P architecture and T4P-plasma membrane interface by SEM after stabilization of the T4P with a monoclonal anti-T4P. (**a**) Example of a single bacterium (false colored in blue) featuring a very dense meshwork of T4P (false colored in yellow) that encloses the bacterial body. Scale bar, 1 µm. (**b**) Example of a pair of bacteria (crop 1) with particularly long T4P (crop 2). Scale bars, 10 µm (large view) and 2 µm (crops). (**c**) Example of a single bacterium where host cell plasma membrane forms protrusions near the bacterial body but not along T4P fiber away from the bacterium, as better seen in the crops. Protr., protrusions. Scale bars, 500 nm (whole picture) and 200 nm (crops). n=2 experiments.

Supplementary Figure 5

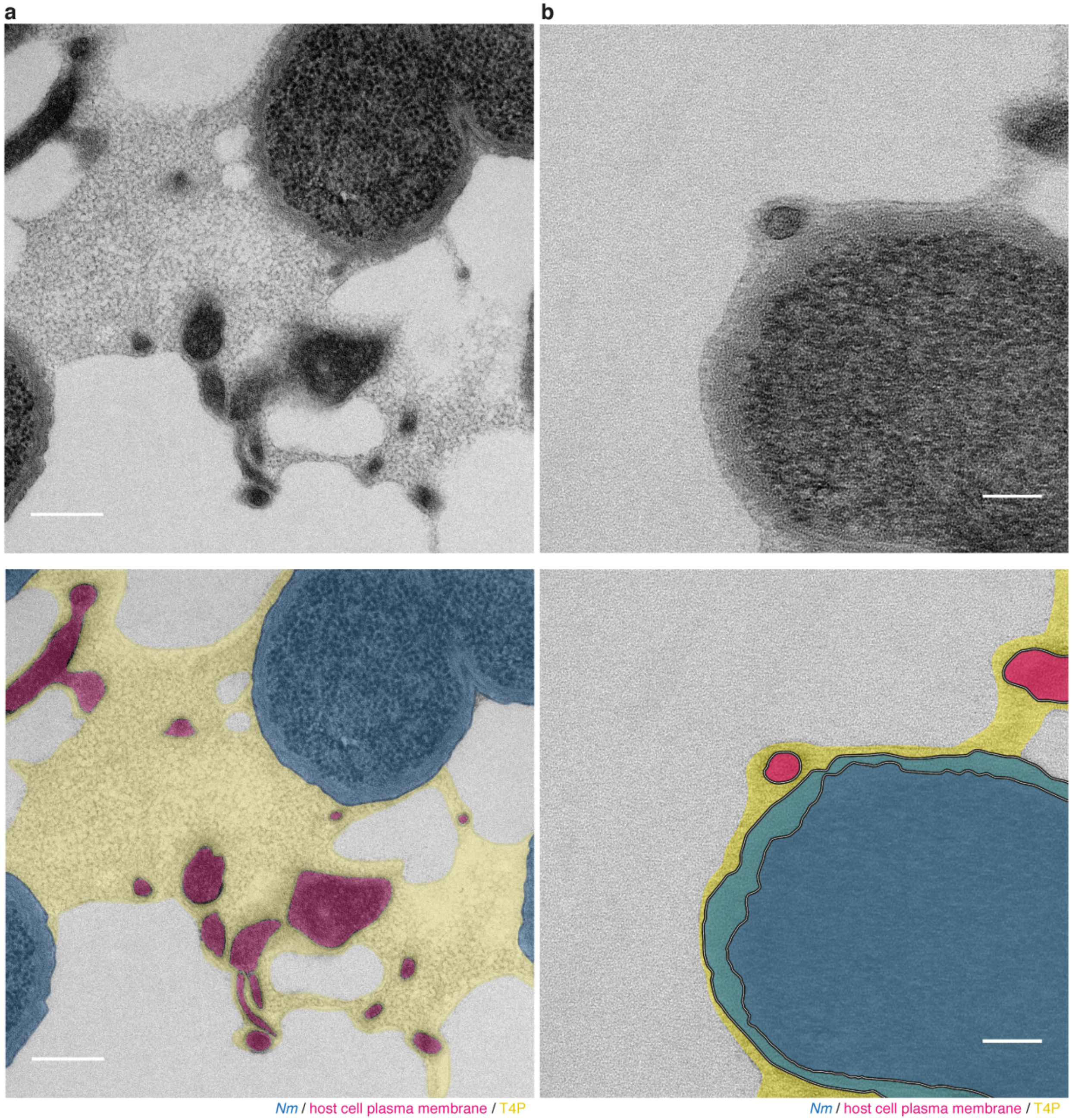

Supplementary Figure 5. Additional examples of the T4P-plasma membrane interface visualized by TEM after HPS-FS. Transmission electron micrographs and colorized micrographs of microcolonies of *Nm* on endothelial cells after 2h of infection, high pressure freezing and freeze substitution. (**a**) Example of a dense meshwork of T4P embedding plasma membrane protrusions. Scale bar, 200 nm. (**b**) Example of a protrusion that lies in a layer of T4P at the periphery of a bacterium with no neighboring bacteria. Scale bar, 100 nm. Representative of multiple microcolonies in n=1 experiment.

Supplementary Figure 6

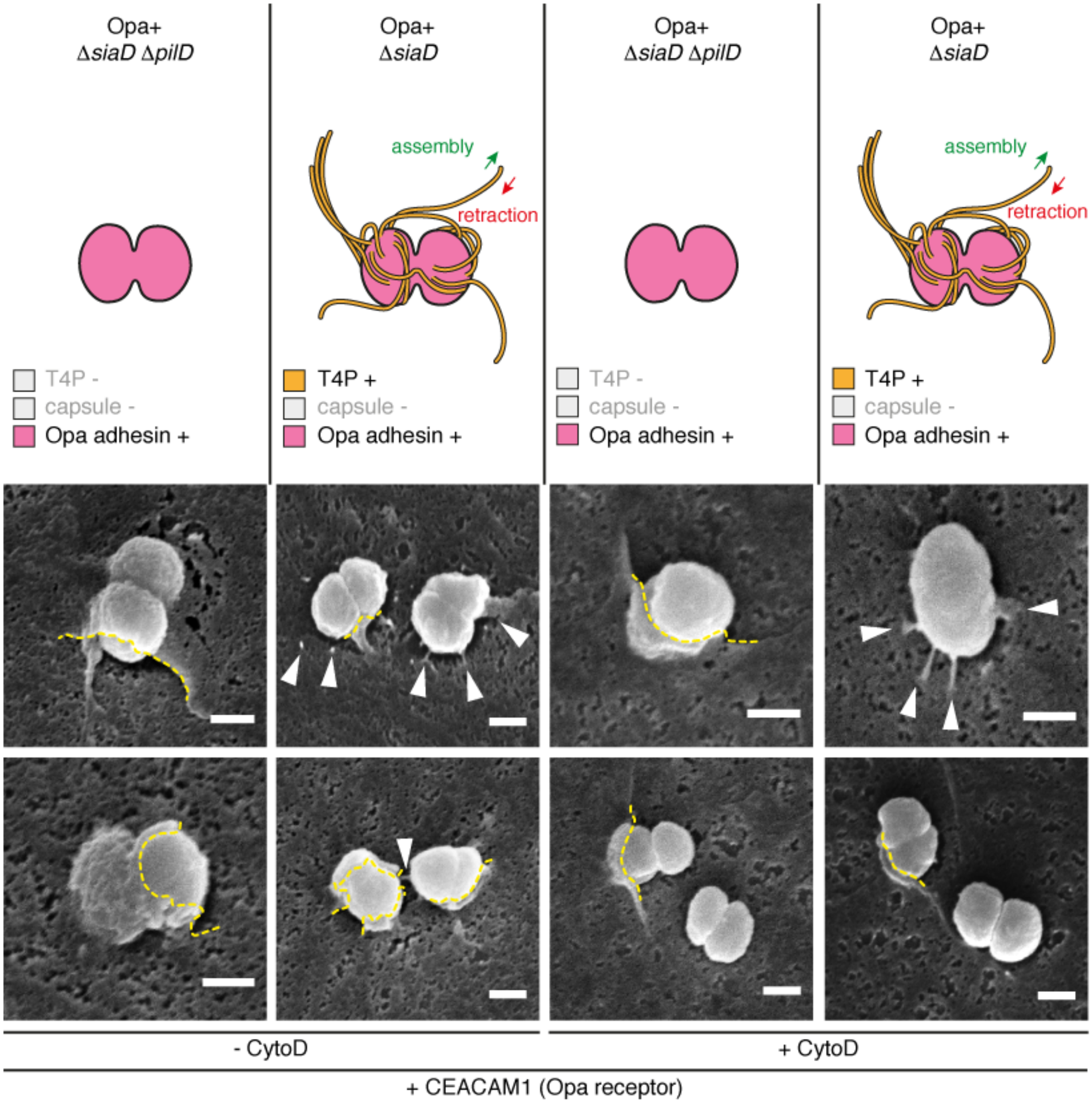

Supplementary Figure 6. Adhesion via non-fibrillar adhesins leads to plasma membrane remodeling as a cup-like structure. Bacteria expressing the alternative Opa outer membrane adhesion elicit plasma membrane remodeling in a cup-like fashion in cells expressing the Opa receptor CEACAM1. Re-expression of T4P in the same genetic background leads to plasma membrane remodeling as a mix of discrete protrusions and incomplete cups. Depolymerization of the F-actin cytoskeleton with cytochalasin D (CytoD) prior to bacterial adhesion does not inhibit plasma membrane remodeling driven by adhesion to either Opa or T4P adhesins. Scale bars, 500 nm. n=1 experiment.

Supplementary Figure 7

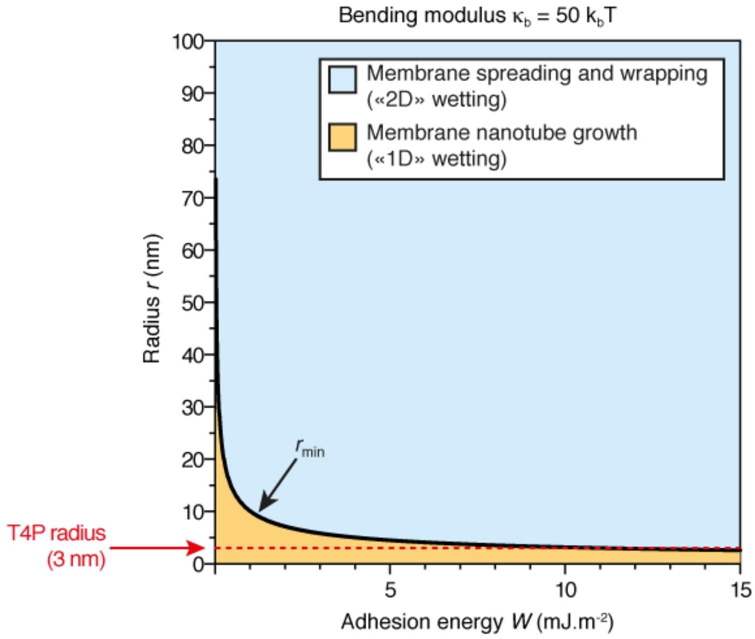

Supplementary Figure 7. Theoretical prediction of "1D" and "2D" wetting regimes for the spreading of a cell membrane on an adhesive nanofiber. Phase diagram of a membrane bilayer spreading on a fiber versus nanofiber radius r and adhesion energy W. The black line corresponds to r_{min} and separates the "2D" (blue region) and "1D" (yellow region) membrane wetting regimes. Here, the bending modulus $\kappa_b \approx 50 k_B T$ is the one found in live cells[1]. The radius of a T4P fiber yields a theoretical minimal adhesion energy of 10 mJ.m^{-2} for "2D" cell membrane wetting to occur.

Supplementary Figure 8

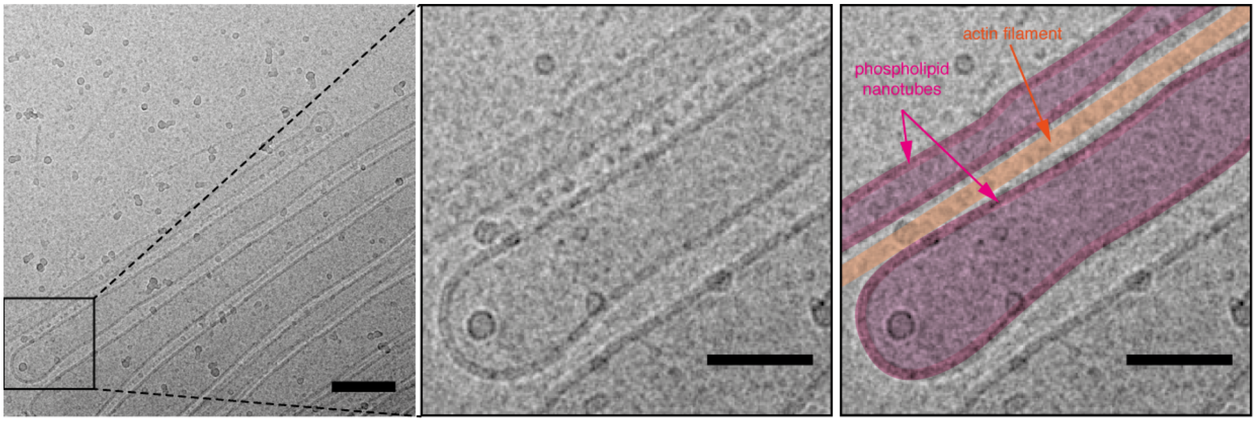

Supplementary Figure 8. Cryo-electron microscopy of vesicles on nanofibers experiment. Here vesicles containing biotinylated phospholipids were mixed with biotinylated F-actin fibers decorated with NeutrAvidin, and imaged by cryo-EM (left). Membrane nanotubes were visibly aligned along individual F-actin filaments instead of wrapping around them (middle and right panels). Scale bars, 100 nm (left) and 50 nm (middle and right panels).

Supplementary References

1 Mohandas, N. & Evans, E. Mechanical-Properties of the Red-Cell Membrane in Relation to Molecular-Structure and Genetic-Defects. *Annu Rev Bioph Biom* **23**, 787-818, doi:DOI 10.1146/annurev.bb.23.060194.004035 (1994).
