# Supplementary material for: Adhesion to nanofibers drives cell membrane remodeling through one-dimensional wetting
Source: Nat Commun. 2018 Oct 25;9:4450. doi: 10.1038/s41467-018-06948-x (PMC6202395; doi:10.1038/s41467-018-06948-x)
Supplement: Supplementary file 3 — Description of Additional Supplementary Files [file 41467_2018_6948_MOESM3_ESM.pdf]

Description of Additional Supplementary Files

File Name: Supplementary Movie 1

Description: Oblique illumination live cell imaging of a HUVEC expressing the membrane marker GFP-F (inverted contrast) infected by Nm-iRFP. A single focal plan is shown. Plasma membrane protrusions from the host cell are visible as discrete bright dots surrounding the bacterial bodies. Scale bar, 10 µm. Representative of several events in n>10 independent experiments.

File Name: Supplementary Movie 2

Description: Oblique illumination live cell imaging of a HUVEC expressing the membrane marker GFP-F and LifeAct-mCherry infected by Nm-iRFP. A single focal plan is shown. Plasma membrane protrusions from the host cell are visible as discrete bright dots surrounding the bacterial bodies. No accumulation of LifeAct-mCherry is observed. Scale bar, 2 µm. Representative of several events in n=2 independent experiments.

File Name: Supplementary Movie 3

Description: Oblique illumination live cell imaging of a micropatterned HUVEC expressing the membrane marker PM-EGFP (inverted contrast) and infected by Nm-iRFP. A single focal plan is shown. Plasma membrane protrusions can be followed over two bacterial divisions. Scale bar, 10 µm. Representative of n=2 independent experiments.

File Name: Supplementary Movie 4

Description: Oblique illumination live cell high speed imaging of a HUVEC expressing GFP-F (inverted contrast) infected with a pre-formed bacterial aggregate of Nm-iRFP (not visible on the movie, please refer to Fig. 2). A single focal plan is shown. The plasma membrane protrusions barely move over 10 seconds, except at the aggregate periphery. Scale bar, 5 µm. Representative of several events in n>10 independent experiments.

File Name: Supplementary Movie 5

Description: Oblique illumination live cell high speed imaging of a HUVEC expressing GFP-F (inverted contrast) infected with wild-type Nm-iRFP bacteria. In this experimental setting, only one channel can be recorded at high speed. The position of 3 bacteria, denoted by a yellow ellipse, was assessed before recording of the GFP-F channel. Plasma membrane protrusions from the host cell are visible as discrete bright dots. Scale bar, 2 µm. Representative of several events in n=10 independent experiments.

File Name: Supplementary Movie 6

Description: Oblique illumination live cell high speed imaging of a HUVEC expressing GFP-F and treated for 20 min with 100nM cytochalasin D (inverted contrast) infected with Nm-iRFP bacteria. In this experimental setting, only one channel can be recorded at high speed. The position of 2 bacteria, denoted by a yellow ellipse, was assessed before recording of the GFP-F channel. Plasma membrane protrusions from the host cell are visible as discrete bright dots. Scale bar, 2 µm. Representative of several events in n=3 independent experiments.

File Name: Supplementary Movie 7

Description: Oblique illumination live cell high speed imaging of a HUVEC expressing GFP-F (inverted contrast) infected with pilT-iRFP bacteria which are deficient for T4P retraction. In this experimental setting, only one channel can be recorded at high speed. The position of 2 bacteria, denoted by a yellow ellipse, was assessed before recording of the GFP-F channel. Plasma membrane protrusions from the host cell are visible as discrete bright dots. Scale bar, 2 µm. Representative of several events in n=3 independent experiments.
