# Supplementary material for: Adhesion to nanofibers drives cell membrane remodeling through one-dimensional wetting
Source: Nat Commun. 2018 Oct 25;9:4450. doi: 10.1038/s41467-018-06948-x (PMC6202395; doi:10.1038/s41467-018-06948-x)
Supplement: Supplementary file 2 — Peer Review File [file 41467_2018_6948_MOESM2_ESM.pdf]

Editorial Note: This manuscript has been previously reviewed at another journal that is not operating a transparent peer review scheme. The manuscript was considered suitable for publication without further review at *Nature Communications.*
